# TRPV1 Channel Contributes to the Behavioral Hypersensitivity in a Rat Model of Complex Regional Pain Syndrome Type 1

**DOI:** 10.3389/fphar.2019.00453

**Published:** 2019-04-26

**Authors:** Qimiao Hu, Qiong Wang, Chuan Wang, Yan Tai, Boyu Liu, Xiaomei Shao, Jianqiao Fang, Boyi Liu

**Affiliations:** ^1^Department of Neurobiology and Acupuncture Research, The Third Clinical Medical College, Zhejiang Chinese Medical University, Key Laboratory of Acupuncture and Neurology of Zhejiang Province, Hangzhou, China; ^2^Department of Pharmacology, Hebei Medical University, Shijiazhuang, China; ^3^Academy of Chinese Medical Sciences, Zhejiang Chinese Medical University, Hangzhou, China

**Keywords:** pain, CRPS-I, TRPV1, dorsal root ganglion neurons, glia

## Abstract

Complex regional pain syndrome type 1 (CRPS-I) is a debilitating pain condition that significantly affects life quality of patients. It remains a clinically challenging condition and the mechanisms of CRPS-I have not been fully elucidated. Here, we investigated the involvement of TRPV1, a non-selective cation channel important for integrating various painful stimuli, in an animal model of CRPS-I. A rat model of chronic post-ischemia pain (CPIP) was established to mimic CRPS-I. TRPV1 expression was significantly increased in hind paw tissue and small to medium-sized dorsal root ganglion (DRG) neurons of CPIP rats. CPIP rats showed increased TRPV1 current density and capsaicin responding rate in small-sized nociceptive DRG neurons. Local pharmacological blockage of TRPV1 with the specific antagonist AMG9810, at a dosage that does not produce hyperthermia or affect thermal perception or locomotor activity, effectively attenuated thermal and mechanical hypersensitivity in bilateral hind paws of CPIP rats and reduced the hyperexcitability of DRG neurons induced by CPIP. CPIP rats showed bilateral spinal astrocyte and microglia activations, which were significantly attenuated by AMG9810 treatment. These findings identified an important role of TRPV1 in mediating thermal and mechanical hypersensitivity in a CRPS-I animal model and further suggest local pharmacological blocking TRPV1 may represent an effective approach to ameliorate CRPS-I.

## Introduction

Complex regional pain syndrome (CRPS) is a severe and debilitating pain condition which can be induced by surgery, fractures, limb trauma, ischemia or nerve lesion (Goh et al., 2017; Birklein et al., 2018). Epidemiological studies estimated an overall incidence rate of CRPS was 26.2/100,000 person years (de Mos et al., 2007). CRPS can develop into chronic condition which severely affects the daily activity and life quality of the patients (Urits et al., 2018). CRPS can be further divided into two subtypes: type-I without identifiable nerve injury and type-II with identifiable nerve injury (Urits et al., 2018). CRPS-I is usually initiated after an initial noxious event and is accompanied with edema, changes in skin blood flow as well as thermal and mechanical hyperalgesia/allodynia in the affected area (Shah and Kirchner, 2011; Vieira et al., 2017). Physiotherapy, sympathetic blockade, corticosteroids, and non-steroidal anti-inflammatory drugs are available treatment options for CRPS-I (Hord and Oaklander, 2003). However, all of these options showed inadequate therapeutic effects on CRPS-I, rendering it a clinically difficult to treat pain condition.

The mechanisms underlying CRPS-I still remain largely unknown. Chronic post-ischemia pain (CPIP) rat model is a well-recognized animal model of CRPS-I, which reproduces peripheral pathology of CRPS-I via ischemia/reperfusion of the hind paws of rats (Coderre et al., 2004). The CPIP model induces early hyperemia and edema, which are followed by chronic neuropathic-like pain symptoms, including spontaneous pain, long-term mechanical and thermal hypersensitivities (Coderre et al., 2004; Luo et al., 2016; Vieira et al., 2017; Garrido-Suarez et al., 2018; Tang et al., 2018). These symptoms recapitulate the typical features of CRPS-I in human patients. By means of this model, several mechanisms, including central glial activation, central pain sensitization, reactive oxygen species increase and activation of peripheral TRPA1, etc. have been proposed to contribute to CRPS-I (Klafke et al., 2016; Kim et al., 2017;Yeo et al., 2017; Tang et al., 2018).

TRPV1 is a non-selective cation channel exclusively expressed in nociceptive primary sensory neurons (Caterina and Julius, 2001). It is a polymodal channel which responds to varies physical and chemical stimuli, including heat, acid pH and mechanical stimulus (Jardin et al., 2017). It is also the principal detector of noxious heat in the peripheral nervous system and plays an important role in mediating thermal hyperalgesia (Caterina et al., 2000; Davis et al., 2000). Genetic ablation or pharmacological blockage of TRPV1 significantly alleviates pain responses in animal models of chronic pain conditions (Szabo et al., 2005; Vilceanu et al., 2010; Wu et al., 2013; Chung et al., 2016).

In order to study the mechanisms underlying CRPS-I, we established the rat model of CPIP. We examined the expression of TRPV1 in peripheral tissue and DRG neurons of CPIP model rats and we studied whether CPIP model could induce peripheral sensitization of TRPV1 channel and enhance DRG neuron excitability. Then we examined the therapeutic effects of locally applied TRPV1 specific antagonist AMG9810 on pain responses of CPIP model rats. Lastly, we explored the effects of AMG9810 on DRG neuron hyperexcitability and spinal glial activation induced by CPIP. Our results demonstrate that TRPV1 plays an important role in mediating the behavioral hypersensitivity of CPIP model rats via promoting peripheral nociceptor activity and spinal glial activation. Pharmacological blockage of TRPV1 may provide an effective therapeutic approach to ameliorate pain responses of CRPS-I patients.

## Materials and Methods

### Animals

Male Sprague-Dawley (SD) rats (8–10 weeks, 300–320 g) were purchased from Shanghai Laboratory Animal Center, Chinese Academy of Sciences and housed in the Laboratory Animal Center of Zhejiang Chinese Medical University accredited by the Association for Assessment and Accreditation of Laboratory Animal Care (AAALAC) under standard environmental conditions (12 h light–dark cycles and 24 ± 2°C). Food and water were provided *ad libitum*. Rats were randomly allocated and 4 rats were housed per cage. The rats were given a minimum of 1 week to adapt to new environment before experiment. All experimental procedures were carried out in accordance with National Institutes of Health guide for the care and use of Laboratory animals (NIH Publications No. 8023, revised 1978) and approved by the Animal Ethics Committee of Zhejiang Chinese Medical University.

### CPIP Rat Model Establishment

Chronic post-ischemia pain was established through exposure to prolonged hind paw ischemia and reperfusion as described previously (Coderre et al., 2004). Anesthesia was induced in all rats with an intraperitoneal injection of 50 mg/kg of sodium phenobarbital and was maintained with an infusion of sodium phenobarbital at 20 mg/kg/hr. An O-ring with 7/32 internal diameter was tightly passed around the right hind limb just proximal to the ankle joint. The O-ring was then cut off 3 h later for reperfusion. Sham rats received the same anesthetic procedure but the ankle was surrounded with a cut O-ring which did not block blood flow.

### Drugs and Administration

AMG9810 (Tocris, United States) was prepared as stock in DMSO and further diluted to 1:1000 in PBS. AMG9810 was applied via intraplantar injection (0.8 μg/25 μl) to the ipsilateral hind paw 40 min before behavioral test. AMG9810 dosage used is based on the effective local dosage reported before (Chung et al., 2015). Sham group rats received vehicle (0.1% DMSO in PBS) injection. Rats received AMG9810 or vehicle treatment daily after CPIP model establishment. All injections were administered by a researcher who was not involved in the behavioral testing.

### Hind Paw Edema

Hind paw edema was evaluated as an increase in paw diameter, measured with a digital caliper and was calculated as the difference between the basal value and the test value observed at different time points after CPIP model establishment.

### Rectal Temperature Assessment

Ambient temperature was automatically regulated at 22 ± 2°C. Rats were anesthetized with isoflurane before the measurement. The isoflurane vaporizer was adjusted to approximately 3–5% for anesthesia induction and approximately 1–3% for maintenance. The rectal thermometer was lubricated with vaseline before insertion. The rectal temperature was measured by gently inserting the digital rectal thermometer to a length of 4–5 cm intrarectal until a stable reading was obtained. After measurements, the probe was cleaned with 70% alcohol. A baseline of rectal temperature was measured before AMG9810 or vehicle treatment (0 h). Then AMG9810 was applied via intraplantar injection into the right hind paw (0.8 μg/paw in 25 μl volume) and control group received vehicle (0.1% DMSO in PBS) treatment. Then rectal temperature was measured at 0.5, 1, 1.5, 2, and 2.5 h after baseline measurement.

### Nocifensive Behavioral Test

Mechanical allodynia: Rats were habituated to the test environment daily for a consecutive 3 days before baseline test. Rats were individually placed in transparent plexiglass chambers on an elevated mesh floor and were habituated for 30 min before test. The mechanical hyperalgesia was determined using a series of von Frey filaments (UGO Basile, Italy) applied perpendicularly to the midplantar surface of the hind paws, with sufficient force to bend the filament slightly for 3–5 s according to methods we previously used (Chai et al., 2018). An abrupt withdrawal of the paw and licking and vigorously shaking in response to stimulation were considered pain-like responses. The threshold was determined using the up-down testing paradigm, and the 50% paw withdrawal threshold (PWT) was calculated by the non-parametric Dixon test [29, 30].

Thermal hyperalgesia: The Plantar Test Apparatus (Ugo Basile, Italy) was used to evaluate thermal hyperalgesia according to previously described. A radiant light beam generated by a light bulb was directed into the right hind paw in order to determine the paw withdrawal latency (the time spent to remove the paw from the stimulus). A 25 s cutoff threshold was set to avoid excessive heating to cause injury. Significant decreases in paw withdrawal latency were interpreted as thermal hyperalgesia. All above behavior tests are conducted by an experimenter blinded to experimental conditions.

### Rotarod Test

Rats were placed on a rotating cylinder with the speed increasing from 5 to 40 rpm in 2 min for four consecutive days for habituation. AMG9810 was applied via intraplantar injection into the right hind paw (0.8 μg/paw in 25 μl volume) for a consecutive of 4 days from Day 1. Control group received vehicle (0.1% DMSO in PBS) treatment. 40 min after each AMG9810 or vehicle treatment, rats were put on rotarod and tested. Tests were repeated 3 times, with 5 min breaks. Falling latency was determined by a stopwatch and averaged. Different batches of rats were used to test mechanical allodynia, thermal hyperalgesia and rotarod test, respectively, in order to avoid the influence of the tests on each other.

### Western Blot

Rats were sacrificed after behavioral test on days 7 and 14, respectively. After anesthetized with pentobarbital (40 mg/kg), the rat was cut open below the diaphragm and the rib cage was cut rostrally on the lateral edges to expose the heart. A small hole was cut in the left ventricle and the needle was inserted into the aorta and clamped, then the right atrium was cut to allow flow. The animal was transcardially perfused with 150 mL cold sterilized 0.9% saline until liver was cleared of blood. Then the ipsilateral L4–6 segments of the DRG, lower part of the spine (T10-L6) and hind paw skin were rapidly removed on ice, and then the spinal cord was flushed out with a forceful injection of ice-cold PBS into the caudal end. Tissues were immediately removed and stored at -80°C. Tissues were homogenized in RIPA buffer (50 mM Tris [pH 7.4], 150 mM NaCl, 1% Triton X-100, 1% sodium deoxycholate, sodium orthovanadate, 0.1% SDS, EDTA, sodium fluoride, leupeptin, and 1 nM PMSF). The homogenate was allowed to rest on ice for 30 min and then centrifuged at 15,000 rpm for 15 min at 4°C and the supernatant was collected. The protein concentration was determined using the BCA method according to the kit’s instruction (Thermo Fisher, United States) and 20 μg of protein was loaded in each lane. Protein samples were separated on 5–10% SDS-PAGE gels and electrophoretically transferred to polyvinyl difluoride (PVDF) membranes (Bio-Rad, United States). The membranes were blocked with 5% non-fat milk at room temperature for 1 h, followed by overnight incubation at 4°C with the following primary antibodies diluted in blocking buffer: anti-TRPV1 rabbit polyclonal antibody (1:1000, Abcam). Subsequently, the immunoblots was incubated with the 2nd antibodies (1: 8000, CST) for 2 h at room temperature. Rabbit anti-GAPDH (HRP Conjugate) (1:1000, Abcam) was used as internal control. The immunoreactivity was detected using enhanced chemiluminescence (BIO-RAD, United States) and visualized with an Image Quant LAS 4000 (EG, United States). The density of each band was measured using Image Quant TL 7.0 analysis software (GE, United States). The mean expression level of the target protein in the animals in the sham group was considered to be 100% and the relative expression level of the target protein in all animals was adjusted as a ratio to the level of the Sham group.

### Immunofluorescence Staining

Rats were deeply anesthetized with sodium pentobarbital (40 mg/kg) and were perfused transcardially with 200 mL 0.9% saline (4°C) followed by 200 ml of 4% formaldehyde. The ipsilateral L4-6 dorsal root ganglia (DRG) and the spinal cord were harvested (contralateral side of the spinal cord was labeled by piercing a needle into the anterior horn) and post-fixed in the same fixative for 4 h (4°C) before transfer to 15%, 30% sucrose for 72 h for dehydration. Several days later, DRG were serially cut into 14 mm thick sections on a frozen microtome (Thermo NX50, United States) and mounted on gelatin-coated glass slides as 6 sets of every 5th serial sections. The spinal cord was also serially cut into 10 mm thick transverse sections were cut on a cryostat to make the slide. All the slides were blocked with 5% normal donkey serum in TBST (with 0.1% Tween-20) for 1 h at 37°C and then incubated overnight with corresponding primary antibodies. The primary antibodies used were rabbit anti-TRPV1 (ab6166, Abcam), mouse anti-GFAP (Abcam), mouse anti-OX42 (Abcam), rabbit anti-NeuN (Abcam). The specificity of the TRPV1 antibody has been validated using TRPV1 knockout mice in a previous study (North et al., 2018). The following day, the sections were rinsed with TBST (6 × 10 min) and incubated for 1 h with a mixture of corresponding secondary antibodies. Fluorescence images were captured by Nikon A1R laser scanning confocal microscope (Nikon, Japan) or Olympus BX61VS virtual slide microscope (Olympus, Japan). For quantitative fluorescence intensity analysis, uniform microscope settings were maintained throughout all image capture sessions. For calculating % of TRPV1 positive neurons, the number of TRPV1 positively stained DRG neurons were divided by the total number of DRG neurons identified by positive NeuN staining. All stained sections were examined and analyzed in a blinded manner. 3–5 images were randomly selected per rat tissue and averaged and then compared according to methods described in our previous studies (Liu et al., 2016a,b).

### DRG Neurons Culture and Patch Clamp Recording

Ipsilateral L4-6 DRG neurons were acutely dissociated as previously described (Liu et al., 2010). Neurons were cultured with DMEM plus 10% fetal bovine serum. The patch-clamp recordings were performed within 48 h. TRPV1 currents were recorded using whole-cell patch clamp technique. Patch pipettes with a resistance of 3–5 MΩ were fabricated from hard borosilicate glasses using a pipette puller (P-97; Sutter Instruments, Novato, CA, United States). Membrane currents were acquired using an Axopatch-200B amplifier (Axon Instruments, Sunnyvale, CA, United States), low-passed at 2 kHz, and sampled at 2–10 kHz. The extracellular solution had the following composition (in mM): NaCl 150, KCl 5, CaCl_2_ 2.5, MgCl_2_ 1, glucose 10, and HEPES 10 (pH 7.4 with NaOH). The internal pipette solution contained (in mM): KCl 140, MgCl_2_ 1, CaCl_2_ 0.5, EGTA 5, HEPES 10, and ATP 3 (pH 7.4 with KOH). Small diameter DRG neurons with Cm < 42 pF were recorded in our test. For voltage clamp, cells were constantly held at -60 mV. Cells were injected with a series of 1 s current from 50 to 500 pA in 50 pA increments (step) or with a linear ramp of current from 0 to 1,000 pA (0.5 s duration) to record action potentials. Data were analyzed with Clampfit 10.2 (Axon Instruments) and Origin 8.0 (Originlab Corporation).

### Statistical Analysis

Statistical analysis was conducted using SPSS 19.0 (SPSS Inc., Chicago, IL, United States). Results were expressed as mean ± SEM. One-way or two-way ANOVA followed by Tukey’s *post hoc* test was used for comparison among groups ≥3. Student’s *t*-test was used for comparisons between two groups. Comparison is considered significantly different if *P* < 0.05.

## Results

### CPIP Model Rats Exhibit Persistent Mechanical Allodynia and Thermal Hyperalgesia

**FIGURE 1:**
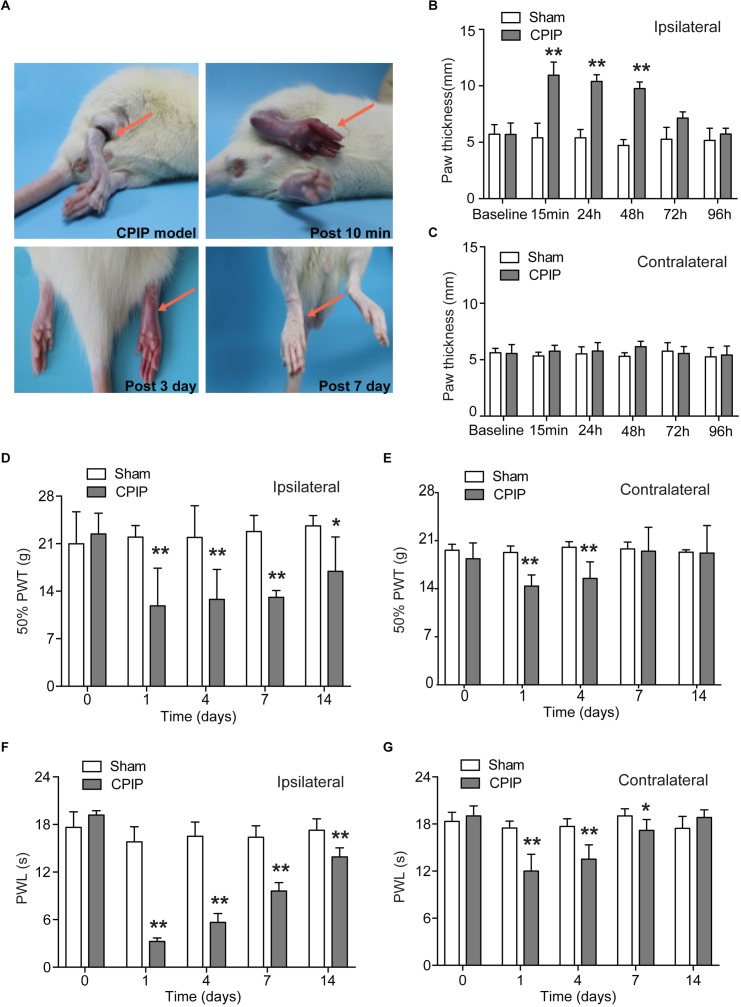
The rat model of chronic post-ischemia pain (CPIP) showed persistent mechanical allodynia and thermal hyperalgesia. **(A)** Representative photographs of rat hind paw taken during CPIP model establishment and 10 min, 3 days, and 7 days after. The red arrow indicates the paw treated with the O ring. **(B,C)** Paw thickness evaluation of ipsilateral **(B)** and contralateral **(C)** paw of Sham and CPIP group rats. **(D,E)** 50% paw withdraw threshold (PWT, for measuring mechanical allodynia) of ipsilateral and contralateral paws of Sham and CPIP group rats. Panel **(D)** shows the PWT of ipsilateral hind paws and panel **(E)** shows the PWT of contralateral hind paws. **(F,G)** Paw withdraw latency (PWL, for measuring thermal hyperalgesia) of ipsilateral and contralateral paws of Sham and CPIP group rats. Panel **(F)** shows the PWL of ipsilateral hind paws and panel (G) shows the PWL of contralateral hind paws. *n* = 7 rats/group. ^∗^*p* < 0.05, ^∗∗^*p* < 0.01 vs. Sham group. Two-way ANOVA followed by Tukey’s *post hoc* test was used for statistical analysis.

We established the rat CPIP model according to methods described before (Coderre et al., 2004). An O-ring tourniquet was used to clamp the right ankle joint for 3 h to block the blood flow to the hind paw. During the procedure, the paw exhibited skin cyanosis, indicating tissue hypoxia (Figure 1A). 10 min after reperfusion, the ipsilateral hind paw, in contrast to contralateral hind paw, was filled with blood and showed edema, demonstrating an intense hyperemia (Figure 1A–C). The edema gradually returned normal 72 h after CPIP model establishment (Figure 1B). 7 days after CPIP model establishment, the ipsilateral paw exhibited dry and shiny appearances (Figure 1A), which were consistent with previous findings (Coderre et al., 2004). We measured the nocifensive behaviors of ipsilateral and contralateral hind paws of CPIP model rats for a consecutive of 14 days. As shown in Figure 1D,E, CPIP model rats developed obvious signs of mechanical allodynia in both ipsilateral and contralateral hind paws, which appeared 1 day after CPIP model establishment (Figure 1D,E). Mechanical allodynia in the ipsilateral paw lasted until 14 days (Figure 1D). We also observed obvious thermal hyperalgesia in both ipsilateral and contralateral hind paws of CPIP model rats, which appeared 1 day after model establishment (Figure 1F,G). Thermal hyperalgesia in the ipsilateral paw lasted until 14 days of the observation period (Figure 1F).

### CPIP Increases TRPV1 Expression in DRG Neurons and Hind Paw Skin Tissues

**FIGURE 2:**
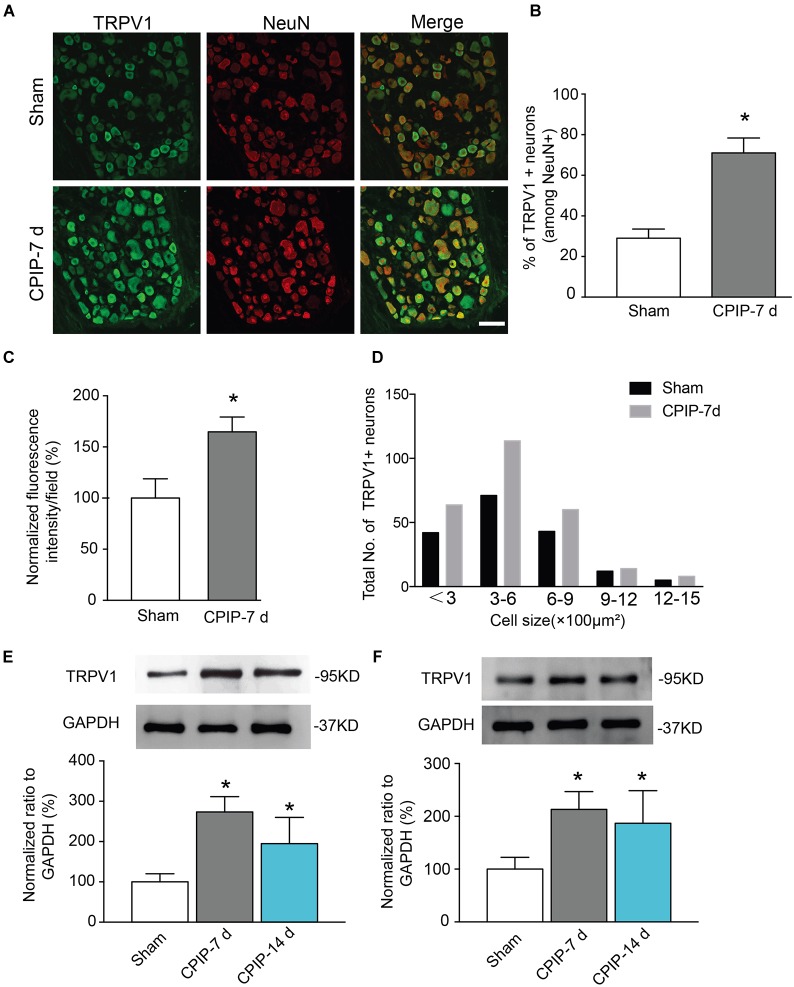
Chronic post-ischemia pain (CPIP) increased TRPV1 expression in rat dorsal root ganglion neurons. **(A)** Representative immunofluorescence images indicating TRPV1 antibody staining of DRGs from Sham and CPIP-7 d group rats. Ipsilateral L4-6 DRGs were collected 7 days post Sham or CPIP model establishment. Areas staining positive for TRPV1 are shown in green. DRGs were co-stained with NeuN antibody (red) to identify DRG neurons. Scale bar indicates 50 μm. **(B)** Summary of the % of TRPV1 positively stained neurons (TRPV1^+^) from each observation field. The total number of DRG neurons per field was calculated based upon positive NeuN (NeuN^+^) staining. **(C)** Summary of the normalized % increase in fluorescence intensity of TRPV1 staining in the observation field as in **A**. The value was normalized to Sham group. **(D)** Size distribution of TRPV1^+^ neurons in DRGs of Sham and CPIP-7 d group rats. Five observation fields from three rats were included in each group. **(E,F)** TRPV1 protein expression in DRGs **(E)** and hind paw skin **(F)** of Sham and CPIP group rats measured with Western blot. Upper panel indicates representative images of TRPV1 and GAPDH protein expression from Sham and CPIP-7 d and 14 d group rats. Lower panel indicates summarized TRPV1 expression normalized to GAPDH. *n* = 5 rats/group. ^∗^*p* < 0.05 vs. Sham group. Student’s *t*-test or one-way ANOVA followed by Tukey *post hoc* test was used for statistical analysis.

TRPV1, a non-selective cation channel, is well known for integrating various painful stimuli in the peripheral sensory neurons. In order to gain insights into the molecular mechanisms underlying CPIP-induced pain, we examined TRPV1 expression in DRGs that innervate the ipsilateral hind paw. Ipsilateral L4-6 DRGs were isolated 7 and 14 days after CPIP model establishment. We detected TRPV1 protein expression in DRG neurons using immunofluorescence staining. All DRG neurons were identified by NeuN staining (Figure 2A). Compared with Sham group, the percentage of TRPV1 positive DRG neurons among all DRG neurons (Neun^+^) were significantly more in CPIP-7 d group than Sham group (Figure 2A,B). The TRPV1 fluorescent staining intensity per observation field was stronger in CPIP-7 d group than Sham group as well (Figure 2C). We analyzed the size distribution of TRPV1^+^ DRG neurons. Immunofluorescence staining revealed that TRPV1 expression was mostly increased in small to medium-sized DRG neurons of CPIP group compared with Sham group (Figure 2D). Western blot further showed that TRPV1 expression was significantly increased in DRGs of CPIP-7 d and CPIP-14 d group compared with Sham group (Figure 2E). We then examined the expression of TRPV1 in ipsilateral hind paw skin tissue of CPIP model rats. As shown in Figure 2F, TRPV1 protein expression in hind paw skin tissue was significantly increased in CPIP-7 d and CPIP-14 d groups compared with Sham group. The above results indicate that CPIP model significantly increased TRPV1 protein expression in both ipsilateral hind paw skin and the innervating peripheral DRG neurons.

### CPIP Increased TRPV1 Channel Current Density in Small Nociceptive DRG Neurons

**FIGURE 3:**
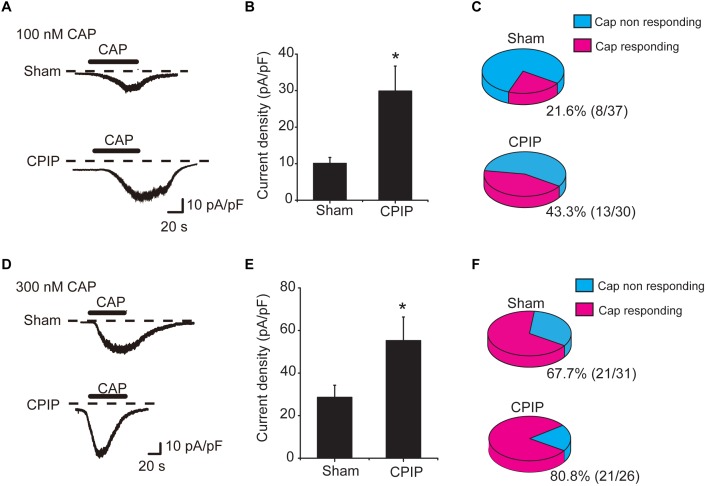
Chronic post-ischemia pain (CPIP) enhanced TRPV1 channel currents in rat DRG neurons. **(A)** Representative current traces showing inward currents induced by TRPV1 agonist capsaicin (CAP, 100 nM) from DRG neurons of Sham and CPIP group, respectively. Current traces were obtained by continuous recording at a holding potential of –60 mV under whole cell voltage clamp. Dotted lines indicate zero current level. Timing of CAP application is indicated by black bars. **(B)** Summary of CAP (100 nM)-induced peak inward current density in DRG neurons from Sham and CPIP group rats. Current amplitude (pA) was normalized with corresponding cell capacitance (pF) to obtain current density (pA/pF). **(C)** Pie charts showing the percentage of CAP (100 nM)-responding neurons (red color) among all tested neurons. The number of neurons tested is as indicated. **(D)** Representative current traces showing inward currents induced by capsaicin (300 nM) from DRG neurons of Sham and CPIP group. **(E)** Summary for the CAP (300 nM)-induced peak inward current density in DRG neurons from Sham and CPIP group rats. **(F)** Pie charts showing the percentage of CAP (300 nM)-responding neurons (red color) among all tested neurons. The number of neurons tested is as indicated. ^∗^*p* < 0.05 vs. Sham group. Student’s *t*-test was used for statistical analysis.

We continued to check whether TRPV1 channel current density was increased in DRG neurons of CPIP model. L4-6 ipsilateral DRG neurons were isolated from both Sham and CPIP group rats 7 days after model establishment and cultured overnight. Whole-cell patch clamp was used to record TRPV1 channel current. We focused on small-sized DRG neurons, which predominately express TRPV1 and play important roles in nociception (Zheng et al., 2012). Using criteria described in previous studies (Abdulla and Smith, 2001; Lopez-Santiago et al., 2006), a DRG neuron was considered small-sized and nociceptive neurons with Cm < 42 pF. Thus, we included DRG neurons showing Cm < 42 pF in our study. After establishing whole-cell recording, DRG neurons were continuously clamped at a holding potential of -60 mV and TRPV1 channel current was induced by bath application of TRPV1 agonist capsaicin. We first tested a relatively small concentration of capsaicin (100 nM). This dosage produces less than half activation of TRPV1 according to our previous report (Zhang et al., 2012). The TRPV1 current amplitude elicited by 100 nM capsaicin was significantly increased in CPIP group than Sham group (Figure 3A). The mean peak current density of TRPV1 elicited by 100 nM capsaicin was 10.1 ± 1.7 pA/pF and 29.9 ± 6.9 pA/pF in Sham and CPIP group, respectively (Figure 3B). Besides, CPIP group showed more percentage of capsaicin responsive neurons than Sham group (43.3% vs. 21.6%, Figure 3C). We also examined TRPV1 channel current in response to a higher concentration of capsaicin (300 nM), which produces almost full activation of TRPV1 as reported (Zhang et al., 2012). TRPV1 current amplitude elicited by 300 nM capsaicin was also significantly increased in CPIP group than in Sham group (Figure 3D). The mean peak current density of TRPV1 elicited by 300 nM capsaicin was 29.8 ± 5.9 pA/pF and 55.2 ± 11.1 pA/pF in Sham and CPIP group, respectively (Figure 3E). The percentage of capsaicin responsive neurons in CPIP group is also higher than Sham group (80.8% vs. 67.7%, Figure 3F). In all, these results demonstrate that CPIP model enhances TRPV1 channel current density and capsaicin responding rate in small-sized nociceptive DRG neurons.

### Pharmacological Blockage of TRPV1 Attenuates Both Thermal and Mechanical Hypersensitivity in CPIP Model Rats

**FIGURE 4:**
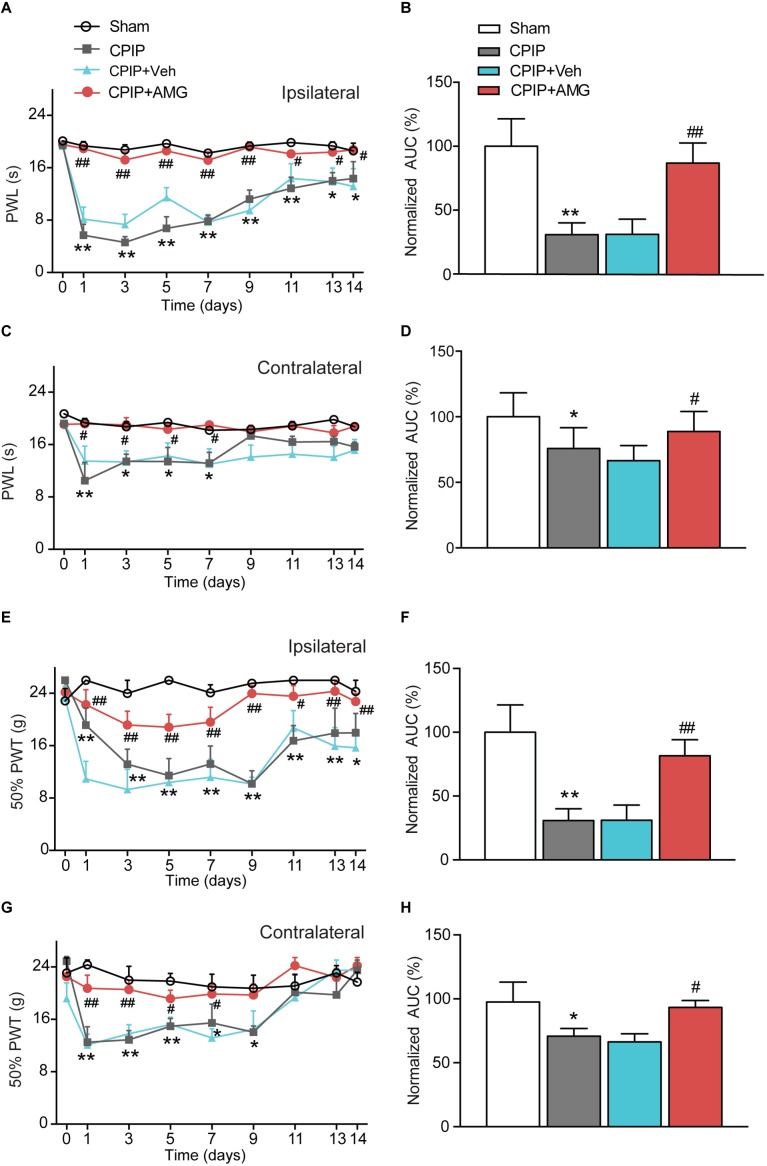
TRPV1 specific antagonist AMG9810 attenuates thermal and mechanical hypersensitivity induced by CPIP model. **(A)** Time course effect of AMG9810 on paw withdraw latency (PWL) of ipsilateral paw of CPIP rats. AMG9810 (0.8 μg/paw, CPIP + AMG) or corresponding vehicle (0.1% DMSO in PBS, CPIP + Veh) was applied via intraplantar injection to ipsilateral hind paws of CPIP rats 40 min before measurement. AMG9810 or vehicle is applied daily after CPIP model establishment. **(B)** Summary of the normalized area under the curve (AUC) as in **A**. **(C)** Time course showing the effects of AMG9810 on paw withdraw latency (PWL) of contralateral paw of CPIP rats. **(D)** Summary of the normalized area under the curve (AUC) as in **C**. **(E)** Time course effect of AMG9810 on 50% paw withdraw threshold (PWT) of ipsilateral paw of CPIP rats. **(F)** Summary of the normalized AUC as in **E**. **(G)** Time course showing the effects of AMG 9810 on 50% PWT of contralateral paw of CPIP rats. **(H)** Summary of the normalized AUC as in **G**. ^∗^*p* < 0.05, ^∗∗^*p* < 0.01 vs. Sham group. ^#^*p* < 0.05, ^##^*p* < 0.01 vs. CPIP + Veh group. *n* = 6 rats/group. Two-way ANOVA followed by Tukey’s *post hoc* test was used for statistical analysis.

We next examined the role of TRPV1 in mediating the nocifensive behavior of CPIP model rats. AMG9810, a potent and specific TRPV1 antagonist (Gavva et al., 2005), was daily applied to the ipsilateral hind paw of CPIP model rat 40 min before behavioral test (0.8 μg/paw, 25 μl, intraplantar injection). The AMG9810 dosage we chose was based upon effective local dosage reported before (Chung et al., 2015). CPIP + Vehicle, Sham + Vehicle, CPIP + AMG, and Sham + AMG groups were established accordingly. As shown in Figure 4A,C, CPIP rats receiving ipsilateral AMG9810 treatment (CPIP + AMG) showed robustly reduced thermal hyperalgesia in both ipsilateral and contralateral hind paws compared with CPIP rats treated with vehicle (CPIP + Veh). Analysis of AUC of Figure 4A,C further indicated an overall inhibition of thermal hyperalgesia produced by accumulated application of AMG9810 compared with vehicle (Figure 4B,D). CPIP rats receiving ipsilateral AMG9810 treatment (CPIP + AMG) at dosage used above also showed significantly attenuated mechanical allodynia in both ipsilateral and contralateral hind paws compared with CPIP rats treated with vehicle (CPIP + Veh) (Figure 4E,G). AUC analysis showed an overall inhibition of mechanical allodynia produced by accumulated application of AMG9810 compared with vehicle (Figure 4F,H).

**FIGURE 5:**
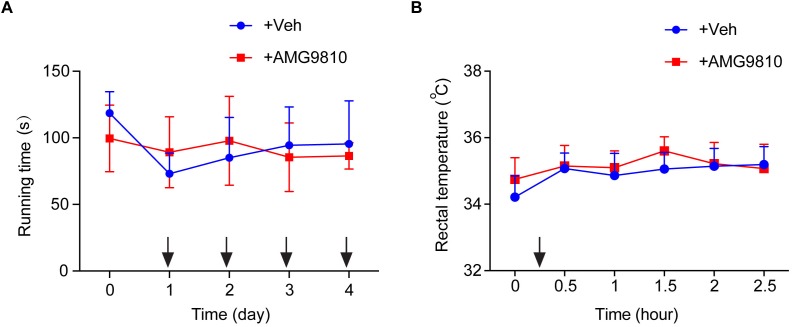
Locally applied low dosage of AMG9810 has no effect on locomotor activity or core body temperature. **(A)** Rat rotarod test to evaluate locomotor activity. AMG9810 was applied via intraplantar injection into the right hind paw (0.8 μg/paw in 25 μl volume) for a consecutive of 4 days from Day 1. Control group received vehicle (0.1% DMSO in PBS) treatment. 40 min after AMG9810 or vehicle treatment, rats were put on rotarod and tested. **(B)** Rectal temperature of rats measured by a digital thermometer. AMG9810 or vehicle was applied at dosages indicated above and applied at time points indicated by the black arrows. *n* = 6 rats/group. Two-way ANOVA followed by Tukey’s *post hoc* test was used for statistical analysis.

We then tested whether local AMG9810 treatment affected motor coordination behavior. AMG9810 was applied via intraplantar injection into the right hind paw (0.8 μg/paw in 25 μl volume, as in Figure 4) for a consecutive of 4 days from Day 1. Control group received vehicle (0.1% DMSO in PBS) treatment. 40 min after each AMG9810 or vehicle treatment, rats were put on rotarod and tested. We found that accumulated local AMG9810 treatment did not affect motor coordination behavior (Figure 5A). Next, we tested whether local AMG9810 treatment affected body temperature. AMG9810 was applied via intraplantar injection into the right hind paw (0.8 μg/paw in 25 μl) and control group received vehicle (0.1% DMSO in PBS) treatment 15 min after baseline temperature measurement (0 h). Then rectal temperature was measured at 0.5, 1, 1.5, 2, and 2.5 h time point. We found that local AMG9810 treatment did not significantly affect body temperature (Figure 5B). In all, the above data demonstrate that pharmacological blocking TRPV1 attenuates both thermal hyperalgesia and mechanical allodynia of CPIP model rats without affecting motor coordination or body temperature.

### Pharmacological Blockage of TRPV1 Reduces the Enhanced Neuronal Excitability of DRG Neurons Induced by CPIP

**FIGURE 6:**
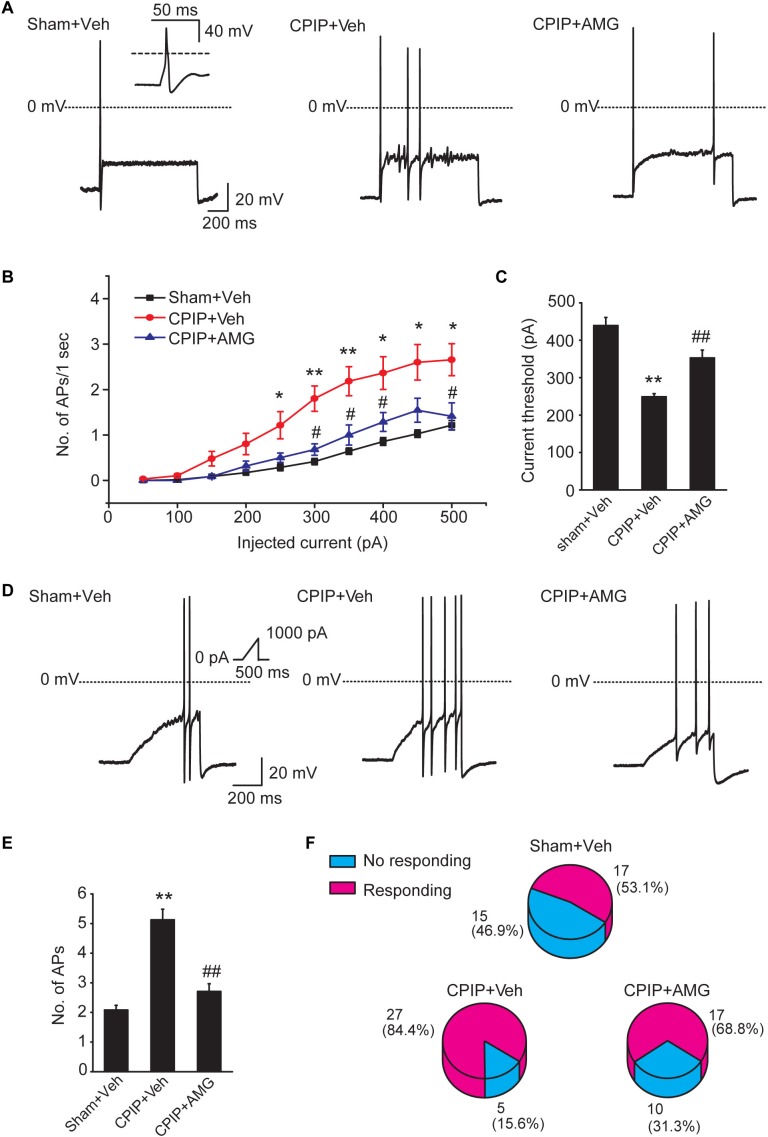
Blocking TRPV1 with AMG9810 decreases the hyperexcitability of nociceptive DRG neurons of CPIP rats. **(A)** Representative current clamp recordings of action potentials of small-sized DRG neurons elicited by 1 s, 400 pA depolarizing current injection. AMG9810 (0.8 μg/paw, CPIP + AMG) or corresponding vehicle (0.1% DMSO in PBS, CPIP + Veh) was applied via intraplantar injection to the ipsilateral hind paws of CPIP rats every day for 7 days. Sham group rats received vehicle injection only. The inset shows the typical action potential which is stretched as recorded in Sham + Veh group. **(B)** Summary of the number of action potentials elicited by depolarizing current steps of DRG neurons from rats of Sham + Veh, CPIP + Veh, and CPIP + AMG groups. *n* = 27 cells/group. Current steps start from 50 to 500 pA, with 50 pA increment, lasting 1 s. **(C)** Summary of the current threshold for eliciting action potentials in DRG neurons of Sham + Veh, CPIP + Veh, and CPIP + AMG groups. **(D)** Representative current clamp recordings of three groups of DRG neurons under ramp current stimulation which starts from 0 to 1,000 pA and lasts 500 ms (see inset). **(E)** Summary of the number of action potentials elicited by ramp current stimulation. **(F)** Pie charts showing the percentage of DRG neurons responding to ramp current stimulation (red color) among all tested neurons. The upper number indicates number of neurons tested and the lower number indicates the percentage within specific group. ^∗^*p* < 0.05, ^∗∗^*p* < 0.01 vs. Sham + Veh group. ^#^*p* < 0.05, ^##^*p* < 0.01 vs. CPIP + Veh group. One-way or two-way ANOVA followed by Tukey’s *post hoc* test was used for statistical analysis.

Chronic pain is usually associated with increased excitability of nociceptive DRG neurons (Wu et al., 2016). To determine whether CPIP can enhance neuronal excitability of DRG neurons and whether TRPV1 is involved in DRG neuron hyperexcitability after CPIP, we studied evoked action potentials (APs) of small-sized DRG neurons (with Cm < 42 pF as described above) via current clamp recording. The averaged Cm of DRG neurons we recorded was around 30 pF (Table 1). Sham + Veh, CPIP + Veh, and CPIP + AMG groups were established accordingly. CPIP model rats were daily treated with AMG9810 (CPIP + AMG) or vehicle (CPIP + Veh) as described in Figure 4A for a consecutive of 7 days. As a control, Sham group rats received vehicle treatment (Sham + Veh). There were no significant differences in resting membrane potentials or after hyperpolarization (AHP) amplitudes of DRG neurons among three groups (Table 1). When stimulated with step current injection, AP firing frequency was significantly increased in CPIP + Veh group compared with Sham + Veh group (Figure 6A,B and Table 1). AMG9810 treatment significantly reduced AP firing frequency in CPIP + AMG group (Figure 6A,B and Table 1). CPIP also significantly lowered the minimal depolarizing current required for evoking APs in DRG neurons and this effect was significantly reversed by AMG9810 treatment (Figure 6C and Table 1). To study the effect of AMG9810 treatment on CPIP-induced neuronal hyperexcitability, we applied a ramp current stimulation protocol under current clamp mode (Figure 6D, shown in inset). As shown in Figure 6D,E, the firing of APs was significantly increased in CPIP + Veh group compared with Sham + Veh group and reduced in CPIP + AMG group (Figure 6D,E and Table 1). We further analyzed the percentage of neurons that fire APs in response to ramp current injection as shown in Figure 6D inset. Data revealed that CPIP + Veh showed more responding rate compared with Sham + Veh group and AMG treatment significantly reduced the responding rate (Figure 6F). Therefore, the above results suggest that CPIP promotes the excitability of nociceptive DRG neurons and pharmacological blocking TRPV1 can reduce the hyperexcitability of DRG neurons induced by CPIP.

**Table 1:** Intrinsic electrogenic properties of small-sized DRG neurons of Sham + Veh, CPIP + Veh, and CPIP + AMG9810 groups of rats.

	Sham + Veh			CPIP + Veh			CPIP + AMG9810		
			
	Mean	*SEM*	*n*	Mean	*SEM*	*n*	Mean	*SEM*	*n*
Input resistance (MΩ)	564.1	40.6	26	537.9	18.2	33	521.7	33.6	33
Capacitance (pF)	30.1	0.4	30	30.3	0.5	31	29.5	0.4	31
RMP (mV)	-65.8	0.9	27	-66.9	1.1	31	-64.9	0.8	32
AP amplitude (mV)	118.5	1.8	32	119.0	2.7	30	120.7	2.2	30
AHP amplitude (mV)	25.1	1.2	32	25.5	0.5	26	24.2	0.9	31
AP frequency (spikes/s, step protocol)	0.9	0.1	30	2.4*	0.4	30	1.3#	0.2	30
AP frequency (spikes/s, ramp protocol)	2.1	0.2	31	5.1**	0.4	30	2.7##	0.3	30
Threshold (pA, step protocol)	439.5	21.6	37	249.0**	8.1	32	352.9##	20.8	32
Threshold (pA, ramp protocol)	726.6	20.5	30	424.4**	23.3	31	529.5##	33.9	30


*AP, action potential; AHP, after hyperpolarization.*

*^∗^p < 0.05, ^∗∗^p < 0.01, compared with sham group; ^#^p < 0.05, ^##^p < 0.01, compared with CPIP + Veh group.*

### Pharmacological Blockage of TRPV1 Reduces Astrocyte and Microglia Activations in Spinal Cord Dorsal Horn of CPIP Model Rats

**FIGURE 7:**
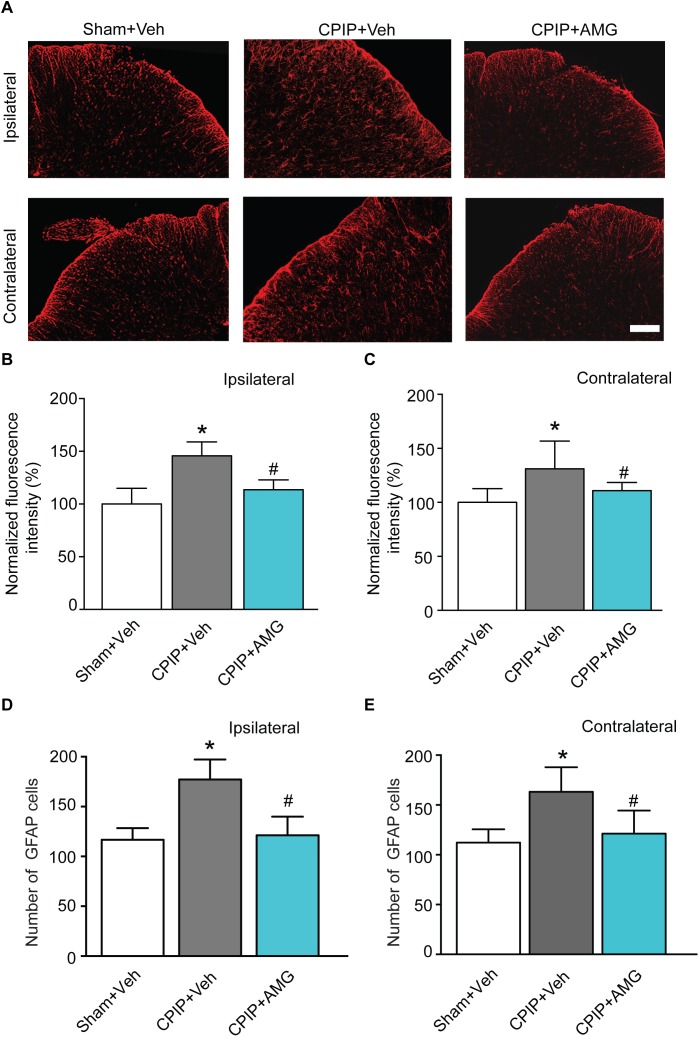
AMG9810 treatment attenuates bilateral astrocyte activation in spinal cord dorsal horn of CPIP rats. **(A)** Ipsilateral (upper panels) or contralateral (lower panels) SCDH stained with GFAP antibody showing astrocytes from Sham + Veh, CPIP + Veh, and CPIP + AMG group. Scale bar = 100 μm. **(B,C)** Summary of the normalized fluorescence intensity (%) of GFAP staining in ipsilateral **(B)** and contralateral **(C)** SCDH. **(D,E)** Summary of the number of astrocytes observed in ipsilateral **(D)** and contralateral **(E)** SCDH. *n* = 4 rats/group. ^∗^*p* < 0.05 vs. Sham + Veh group. ^#^*p* < 0.05 vs. CPIP + Veh group. One-way ANOVA followed by Tukey’s *post hoc* test was used for statistical analysis.

It is known that glial cells in the spinal cord dorsal horn (SCDH) play important roles in the development and maintenance of chronic neuropathic pain (Milligan and Watkins, 2009). Therefore, we proceeded to explore the involvement of TRPV1 in astrocyte and microglia activation in SCDH of CPIP model rats. Sham + Veh, CPIP + Veh, and CPIP + AMG groups were established accordingly. CPIP model rats received intraplantar injection of AMG9810 (CPIP + AMG) or vehicle (CPIP + Veh) to the ipsilateral side daily as described before and sacrificed 7 days later. Sham groups received vehicle treatment (Sham + Veh). We monitored the changes of immunoactivity of GFAP (an astrocyte marker) in SCDH. We observed strong increases in GFAP immunoactivity and the number of GFAP positive cells in CPIP + Veh group compared with Sham + Veh group in both ipsilateral and contralateral sides of SCDH (Figure 7A–E). Ipsilateral AMG9810 treatment significantly attenuated GFAP immunoactivity and the number of GFAP positive cells in both sides of SCDH (Figure 7A–E).

**FIGURE 8:**
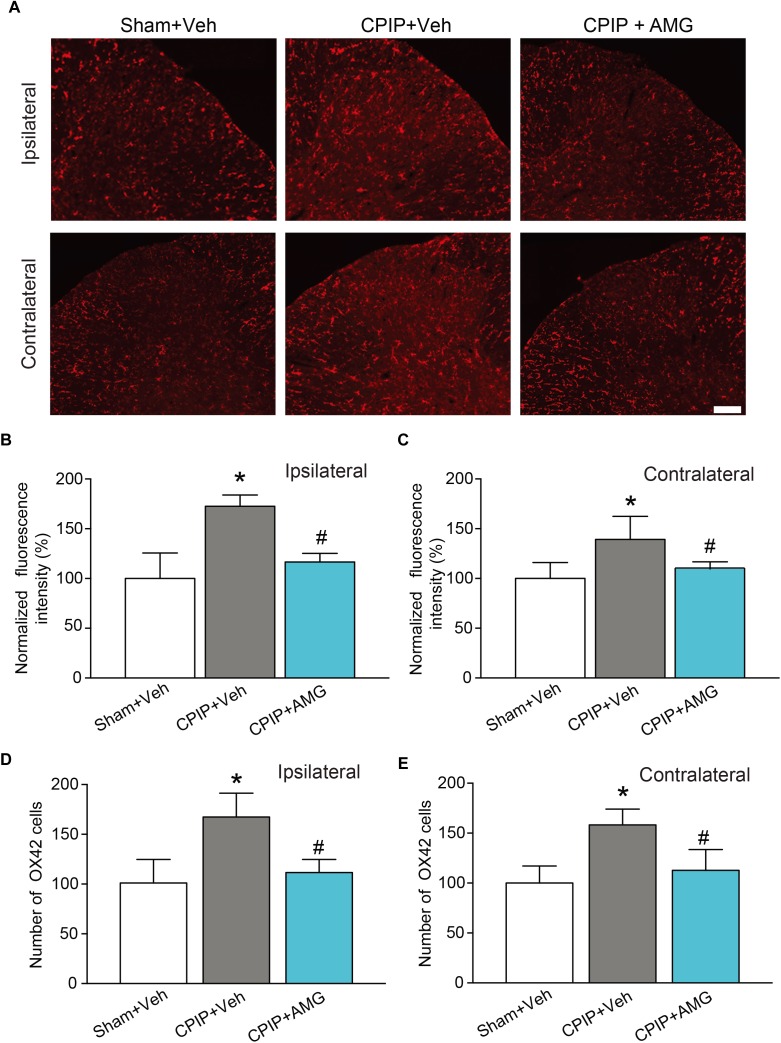
AMG9810 treatment attenuates bilateral microglia activation in spinal cord dorsal horn of CPIP rats. **(A)** Ipsilateral (upper panels) or contralateral (lower panels) SCDH stained with OX42 antibody showing microglia from Sham + Veh, CPIP + Veh, and CPIP + AMG group. Scale bar = 100 μm. **(B,C)** Summary of the normalized fluorescence intensity (%) of OX42 staining in ipsilateral **(B)** and contralateral **(C)** SCDH. **(D,E)** Summary of the number of microglia observed in ipsilateral **(D)** and contralateral **(E)** SCDH. *n* = 4 rats/group. ^∗^*p* < 0.05 vs. Sham + Veh group. ^#^*p* < 0.05 vs. CPIP + Veh group. One-way ANOVA followed by Tukey’s *post hoc* test was used for statistical analysis.

We then examined the changes of immunoactivity of OX42 (a microglia marker) in SCDH. We observed a significant increase of OX42 immunoactivity and the number of OX42 positive cells in CPIP + Veh group compared with Sham + Veh group in both ipsilateral and contralateral side of SCDH, whereas ipsilateral AMG9810 treatment significantly reduced the OX42 immunoactivity and the number of OX42 positive cells in both sides (Figure 8A–E). These results demonstrate that CPIP is accompanied with significant astrocyte and microglia activations in SCDH and pharmacological blocking TRPV1 effectively attenuates astrocyte and microglia activations.

## Discussion

In the present study, we identified an important role of TRPV1 in mediating the behavioral hypersensitivity of CPIP rat model. We found that TRPV1 protein expression was significantly increased in DRG neurons that innervate the hind paw of CPIP rats. Size distribution analysis further revealed that TRPV1 expression was mainly increased in small to medium-sized DRG neurons, which were predominantly comprised by nociceptive neurons. We also observed that the expression of TRPV1 protein in the hind paw skin tissue of CPIP model rats was significantly increased. The tendency of TRPV1 up-regulation in DRGs and hind paw skin tissue correlated well with the development of CPIP-induced thermal and mechanical hypersensitivity throughout the time frame we observed. Up-regulation of TRPV1 in peripheral DRG neurons has been described in some inflammatory and neuropathic pain conditions (Hara et al., 2013; Simonic-Kocijan et al., 2013; Lin et al., 2015). Our patch clamp study further indicated that TRPV1 channel current density was significantly enhanced in small-diameter DRG neurons from CPIP model rats, suggesting that CPIP enhanced the expression of functional TRPV1 channels in the plasma membrane of primary nociceptors. Therefore, the up-regulation of TRPV1 protein expression and channel activity in nociceptive DRG neurons may participate in CPIP-induced pain responses.

AMG9810 is a competitive TRPV1 antagonist with high specificity and potency (Gavva et al., 2005). It effectively blocks TRPV1 activation induced by capsaicin, protons, heat, and endogenous ligands (Gavva et al., 2005). AMG9810 is effective in alleviating thermal and mechanical hyperalgesia and pain responses in several animal models of inflammatory and neuropathic pain, suggesting its utility as a potential analgesic (Wu et al., 2013; Chung et al., 2016; Wang et al., 2018). Unfortunately, systematic AMG9810 treatment (30 mg/kg via intraperitoneal injection) resulted in an increase in body temperature (hyperthermia) in tested animals, a common side effect among all TRPV1 antagonists (Gavva et al., 2007). This unwanted side effect constitutes a hurdle for developing TRPV1 antagonists as potential pain relievers (Gavva, 2008). In our study, AMG9810 was applied via local intraplantar injection instead of systematic administration and a much lower dosage (0.8 μg/site) was used. This dosage, if converted to body weight, is estimated to be 2.8 μg/kg, which is more than 1,000-fold less than the dosage used in previous studies (Gavva et al., 2005; Gavva, 2008). At this dosage, we did not observe any significant increase in body temperature in AMG9810 treated rats. Besides, we found that AMG9810 treated rats still showed response to heat stimulus, with PWLs similar with those of the sham group (Figure 4A). This phenomenon indicates that the therapeutic effect of AMG9810 on thermal hyperalgesia was not simply due to the failure of the treated rat to detect thermal stimulation. This finding is consistent with other studies suggesting that AMG9810 at certain dosage mainly blocks TRPV1-dependent thermal hypersensitivity rather than thermal detection (Wu et al., 2013). Recently, some modality-specific TRPV1 antagonists, which do not affect body temperature and thermal detection, have been developed (Reilly et al., 2012; Brown et al., 2017). Therefore, our study suggests that local application of AMG9810 or the new modality-specific TRPV1 antagonists may be used clinically to relieve CRPS-I related pain without obvious alterations in body temperature and thermal detection. The effectiveness of other more convenient local application routes, such as patch or ointment, is worth of further testing.

One important mechanism through which TRPV1 channel may participate in chronic pain is to promote hyperexcitability of primary nociceptive neurons, which in turn drives central sensitization and chronic pain condition (Chung and Chung, 2002; Berta et al., 2017). In our study, we found that DRG neurons derived from CPIP rats exhibited enhanced excitability compared with sham rats. The hyperexcitability is significantly reduced by *in vivo* TRPV1 blockage. This result suggests that TRPV1 channel is important for enhancing the excitability of DRG neurons of CPIP rats. Nav sodium channels play crucial roles in regulating action potential firing frequency and excitability in DRG neurons (Rush et al., 2007). It has been reported that activation of TRPV1 enhances Nav sodium currents in DRG neurons (Lu et al., 2016). Furthermore, *Trpv1* gene deletion significantly reduced the overexpression of Nav1.7 and 1.8 channels and action potential firings in DRG neurons from chronic pain model animals (Wu et al., 2013; Liao et al., 2017). These findings suggest that TRPV1 can regulate DRG neuron excitability via modulating Nav channel expression and activities. In our study, we found that TRPV1 channel expression and activities in DRG neurons are significantly enhanced in CPIP rats. This effect may result in upregulation of Nav channel expression or activity in DRG neurons, which in turn could produce hyperexcitability in response to current injection recorded by current clamp. Repetitive *in vivo* AMG9810 treatment could block TRPV1 activity, which may reduce the upregulation of Nav channel expression and ameliorate the hyperexcitability of DRG neurons. Nav sodium channel expression is upregulated in several pain conditions, resulting in hyperexcitability of sensory neurons and pain (Black et al., 2004; Liang et al., 2013; Wu et al., 2016). Therefore, further studies will be needed to explore the expression changes of Nav sodium channels and the possible interactions of TRPV1 with Nav sodium channels in DRG neurons from CPIP rats. These studies would allow us to have a better understanding of the mechanisms underlying CPIP-induced DRG neuron hyperexcitability and behavioral hypersensitivity.

Thermal and mechanical hypersensitivities are among the major symptoms affecting CRPS-I patients (Grothusen et al., 2014; Terkelsen et al., 2014; Reimer et al., 2016; Rasmussen et al., 2018). A recent study demonstrated CPIP model rats developed obvious signs of thermal hyperalgesia, which correlates with our present findings (Tang et al., 2018). Moreover, CRPS patients showed obvious increase in capsaicin-induced pain in affected limbs, indicating that TRPV1 function is likely to be up-regulated in CRPS patients (Terkelsen et al., 2014). Our findings further helps to explain the increased capsaicin-induced pain response in CRPS patients. In our study, the behavioral hypersensitivity of CPIP rats gradually returned near normal after 2 weeks of observation. But CRPS-I patients usually suffered a more chronic period of time. Therefore, the results we obtained from the CPIP model may not completely reflect the mechanisms in the chronic phases of CRPS-I patients. Our present results suggest that TRPV1 may participate in early phase of CRPS-I. But it still remains to be investigated whether TRPV1 is involved in chronic phases of CRPS-I in future studies.

Chronic post-ischemia pain model rats developed obvious signs of mechanical allodynia and thermal hyperalgesia in both ipsilateral and contralateral hind paws, a phenomenon similar with human CRPS-I patients showing bilateral hypersensitivity to painful chemical, thermal, and mechanical stimuli (Terkelsen et al., 2014). It is proposed that central sensitization may underlie this generalized bilateral hypersensitivity in CRPS-I (Terkelsen et al., 2014). Glial cells in the SCDH, such as microglia and astrocytes, are activated in response to peripheral painful stimuli and participate in spinal pain signal transmission and central sensitization (Milligan and Watkins, 2009; Yu et al., 2018). Astrocytes and microglia in both ipsilateral and contralateral SCDH are activated in CPIP model rats (Tang et al., 2018). Pharmacological blockage of microglia or astrocytes activation in the spinal cord alleviates CPIP-induced bilateral mechanical allodynia and thermal hyperalgesia, suggesting glial cell activation is directly involved in CPIP model induced bilateral pain responses (Tian et al., 2017; Tang et al., 2018). In our study, we found that ipsilateral hind paw injection of AMG9810 significantly alleviated bilateral hind paw thermal and mechanical hypersensitivity. Immunohistochemistry study further revealed that ipsilateral AMG9810 treatment attenuated astrocyte and microglia activations in bilateral SCDH. Therefore, we propose that blocking TRPV1 attenuates hyperexcitability of nociceptive DRG neurons of CPIP model rats and reduces peripheral pain signal input into SCDH, which in turn results in reduced spinal glia activation, thereby attenuates bilateral thermal and mechanical hypersensitivity.

Epidemiology studies found that the incidence of CRPS-I is higher in female than male patients (Weissmann and Uziel, 2016; Kim et al., 2018). Animal studies also identified that CPIP female mice developed significantly earlier and higher mechanical allodynia in the ischemic hind paw than male mice (Tang et al., 2017). In the past decade, sex differences in pain perception have shown increasing importance in both clinical and experimental studies (Kindler et al., 2011; Boerner et al., 2017). Different mechanisms may underlie pain perception of male and female animals (Sorge et al., 2015). In the present study, we only used male rats to establish CRPS-I model according to previous publications. Further studies will be needed to explore the effects of TRPV1 antagonist on female CRPS-I model animals, which are crucial for future translational study of treatment options for CRPS-I patients.

At present, CRPS-I patients are mainly treated with physiotherapy, sympathetic blockade, corticosteroids, and non-steroidal anti-inflammatory drugs (Hord and Oaklander, 2003). However, all of these options showed only moderate therapeutic effects on CRPS-I, which make CRPS-I a clinically difficult to treat condition. We identified an important role of TRPV1 in mediating the thermal and mechanical hypersensitivities in a CRPS-I rat model and suggest local pharmacological blocking of TRPV1 with AMG9810 or other specific TRPV1 antagonists may help to relieve pain symptoms of CRPS-I patients in early phases.

## Ethics Statement

This study was carried out in accordance with National Institutes of Health guide for the care and use of Laboratory animals (NIH Publications No. 8023, revised 1978) and approved by the Animal Ethics Committee of Zhejiang Chinese Medical University.

## Author Contributions

QH performed the western blot, immunostaining, and behavioral test. QW performed the patch clamp experiments. CW supervised the patch clamp experiments. QH and BoyuL performed the behavioral test. YT, XS, and JF analyzed the data and reviewed the manuscript. BoyiL designed, supervised the study, and wrote the manuscript.

## Conflict of Interest Statement

The authors declare that the research was conducted in the absence of any commercial or financial relationships that could be construed as a potential conflict of interest.
